# Conversational Agents for Body Weight Management: Systematic Review

**DOI:** 10.2196/42238

**Published:** 2023-05-26

**Authors:** Eunyoung Noh, Jiyoon Won, Sua Jo, Dae-Hyun Hahm, Hyangsook Lee

**Affiliations:** 1 Department of Medical Science of Meridian College of Korean Medicine Kyung Hee University Seoul Republic of Korea; 2 Department of Meridian & Acupoint, College of Korean Medicine Dong-eui University Busan Republic of Korea; 3 Department of Physiology College of Medicine Kyung Hee University Seoul Republic of Korea

**Keywords:** conversational agent, chatbot, obesity, weight management, artificial intelligence, behavioral therapy

## Abstract

**Background:**

Obesity is a public health issue worldwide. Conversational agents (CAs), also frequently called chatbots, are computer programs that simulate dialogue between people. Owing to better accessibility, cost-effectiveness, personalization, and compassionate patient-centered treatments, CAs are expected to have the potential to provide sustainable lifestyle counseling for weight management.

**Objective:**

This systematic review aimed to critically summarize and evaluate clinical studies on the effectiveness and feasibility of CAs with unconstrained natural language input for weight management.

**Methods:**

PubMed, Embase, the Cochrane Library (CENTRAL), PsycINFO, and ACM Digital Library were searched up to December 2022. Studies were included if CAs were used for weight management and had a capability for unconstrained natural language input. No restrictions were imposed on study design, language, or publication type. The quality of the included studies was assessed using the Cochrane risk-of-bias assessment tool or the Critical Appraisal Skills Programme checklist. The extracted data from the included studies were tabulated and narratively summarized as substantial heterogeneity was expected.

**Results:**

In total, 8 studies met the eligibility criteria: 3 (38%) randomized controlled trials and 5 (62%) uncontrolled before-and-after studies. The CAs in the included studies were aimed at behavior changes through education, advice on food choices, or counseling via psychological approaches. Of the included studies, only 38% (3/8) reported a substantial weight loss outcome (1.3-2.4 kg decrease at 12-15 weeks of CA use). The overall quality of the included studies was judged as low.

**Conclusions:**

The findings of this systematic review suggest that CAs with unconstrained natural language input can be used as a feasible interpersonal weight management intervention by promoting engagement in psychiatric intervention-based conversations simulating treatments by health care professionals, but currently there is a paucity of evidence. Well-designed rigorous randomized controlled trials with larger sample sizes, longer treatment duration, and follow-up focusing on CAs’ acceptability, efficacy, and safety are warranted.

## Introduction

### Background

Obesity, nearly tripled between 1975 and 2016 [1], causes adverse health effects associated with various comorbidities, including type 2 diabetes mellitus [2], cardiovascular diseases [3], cancers [4], and musculoskeletal problems [5]. According to estimates, the cost of treating obesity increased from US $124.2 billion in 2001 to US $260.6 billion in 2016, and in the United States, persons with obesity spend approximately US $2505 (or 100%) more on medical expenses than people of normal weight [6]. Dietary changes (ie, caloric restriction; changes in fat, protein, and carbohydrate intake rates; or taking macronutrient substitutes) as well as exercise and behavioral modification (ie, gradually changing eating and physical activity habits) are generally recommended to manage or prevent obesity-related problems [7]. In the short term, combining diet and exercise can result in substantial weight loss, and behavioral adjustment appears to prevent weight gain [7]. Despite the benefits of combining interventions, generally known as lifestyle modification (LM) [8], primary medical institutions only deliver LM to a limited extent [9]. Lack of training, limited counseling time, and additional costs associated with more frequent visits are all possible factors for providing less LM counseling [9]. In this context, digital health interventions (DHIs) have been proposed as an alternative approach to providing LM [10], which allows for the self-management of behavioral programs, improves patient adherence, and lowers costs [11-13]. However, a recent systematic review indicated that social functions similar to the in-person experience, such as personalization and conversational components, are required in DHIs to keep people engaged for the longer term and enhance outcome effectiveness [12,14].

Conversational agents (CAs), often called chatbots, are computer programs that replicate human dialogue (ie, interpret user inputs and respond appropriately through textual or spoken language) [15]. The emergence of new technologies such as artificial intelligence (AI), machine learning, and natural language processing [16] allows users to communicate with CAs using unconstrained natural language input [17,18], which enables more complex and flexible conversations [19]. As a result, CAs have been suggested as a viable alternative to face-to-face lifestyle counseling for weight management and to overcome DHIs’ limitations [20]. So far, published systematic or scoping reviews of CAs have mainly focused on health care in general [21,22] or mental health [16,23-26]. More recently, 2 reviews on CAs for weight loss behavior were published: one systematic review included various chatbot types, and the outcomes of interest were physical activity or dietary change for general health improvement [27], and the other scoping review intended to focus on AI CAs for weight loss, but the included studies adopted diet and exercise for general health improvement in heterogeneous populations (eg, physical activity for patients with cancer) and only constrained conversation with AI CAs was available [28].

Although personalized, humanlike, unconstrained CAs provide long-term efficacies that enable users to better engage in conversations and adhere to customized intervention messages, previous reviews have included heterogeneous CAs or participants, the intervention’s goal was not solely weight management, and there were few unconstrained CAs [27,28]. Considering the rapid advances in technology in this field, an updated systematic review with specific questions is needed.

### Objectives

In this context, this systematic review was conducted to critically evaluate the effectiveness of CAs using unconstrained natural language input on weight loss or obesity-related outcomes (ie, physical activity and dietary change) and their feasibility in clinical practice.

## Methods

### Search Strategy

The protocol for this systematic review was registered with the Research Registry system (Review Registry Unique Identifying Number: reviewregistry960). This systematic review was compliant with the PRISMA (Preferred Reporting Items for Systematic Reviews and Meta-Analyses) statement (Multimedia Appendix 1 [29]). PubMed, Embase, the Cochrane Library (CENTRAL), PsycINFO, and ACM Digital Library were searched on December 26, 2022, from the inception of each database to identify studies on the application of CAs in weight management. Hand searching was performed for relevant studies in the reference lists of the included studies. An extensive list of 28 search terms was used: (1) various synonyms of CA and (2) obesity-related terms (Multimedia Appendix 2). The search terms were reviewed by an independent senior information specialist before conducting the search.

### Eligibility Criteria

Studies were included if the user communicated directly with a CA for the purpose of weight management; no restrictions on participants were imposed (ie, overweight, obese, or healthy volunteers) if they interacted with a CA intended for weight management. As the definition of CAs differed and there was a lack of consensus, CAs in this systematic review were sought if digital tools mimicked humanlike behaviors and provided a task-oriented framework with participation in conversation [16]. CAs that used any unrestricted natural language were also included as they were considered more interactive than simple CAs with predefined answer options [22,23]. All types of digital delivery tools were included, such as text-based chatbots (casual conversation delivered verbally or in text), embodied CAs (ECAs; animated virtual characters that enable face-to-face interaction) [20], or CAs within virtual reality [23]. There were no restrictions on the type of dialogue initiative or input or output modality. Regarding primary outcomes, studies that reported body weight (measured in kilograms or pounds), BMI, or waist circumference measured as an absolute change or percentage change from the baseline were included. Regarding secondary outcomes, any obesity-related outcomes (eg, weight loss–related, physical activity–related, diet-related, any measure of energy expenditure, satisfaction, usability, effect modifiers or confounders for adherence, psychological or behavior changes, health-related quality of life, safety, process measures, and cost-effectiveness) were sought. Any type of clinical research was considered only if it was published in a peer-reviewed journal, but no restrictions on study design, language, or publication date were imposed. Studies were excluded if they were reviews, news articles, or conference abstracts. Studies were also excluded if they did not use CAs for weight management; used *Wizard of Oz* methods, in which the dialogues were generated by an unseen human operator, only predetermined, and not generated in response to user input [23]; focused on the technical function and development of the CAs; or did not report the outcome of interest in the systematic review.

### Study Selection and Data Extraction

To determine the eligibility of the studies, they were first screened based on the titles and abstracts to determine whether the full texts should be further evaluated. Full texts were then obtained, read in full, and excluded with specific reasons. The areas in which CAs contributed to weight management and the strategy of CAs for weight management were identified and divided into two categories: (1) study characteristics and (2) CA characteristics. Regarding study characteristics, predefined extraction forms were used to collect relevant information, which included study design, year and country of study, first author, sample size, participants (including age, sex, degree of obesity, and recruitment site), intervention duration, control group description if relevant, CA contributing to conventional weight management program (ie, dietary modification, increased physical activity, or behavioral modification), and study outcomes. Regarding the characteristics of CAs, delivery device or platform, type of CA (ie, text-based or ECA), input or output modality (written or spoken), whether the CA mimicked practice (presenting what aspects of human health care experts CAs were designed to emulate), theories integrated into CAs, commercialization, and personalization were extracted. If the CA was capable of personalization, personalization methods were also extracted (eg, processing previous interactions with users or users entering information when setting up) [28]. The articles were independently screened and extracted by 2 reviewers, and any disagreements were resolved through discussion. To address missing or unclear information in the study selection and data extraction, several assumptions were made, although it was best recommended to contact the authors. Even if the term *CA* or *chatbot* was not used, it may be reasonable to assume that automated interactive digital tools with participation in conversation were regarded as CAs. If a reduction in body weight was reported, we assumed that the CA was used for weight management and participants had an intention of weight management. A variety of adherence measurements that could be evaluated were considered, for example, session attendance, retention or dropout rates, or the frequency and duration of CA use (eg, logging into CAs or number of days in which calories were recorded) [30,31]. For multiple treatment trials, the treatment arms were combined, and for crossover trials, the first follow-up information was used.

### Quality Assessment of the Included Studies

The included studies were assessed by 2 reviewers to evaluate their quality, and disagreements were resolved through discussion. For randomized controlled trials (RCTs), the revised Cochrane risk-of-bias tool for randomized trials was used [32]. For the rest of the study designs, such as observational studies, economic evaluations, diagnostic studies, qualitative studies, and clinical prediction rules, the Critical Appraisal Skills Programme checklist was used [33].

### Data Synthesis

A narrative systematic review was conducted for all the included studies because of the expected heterogeneity of study characteristics, CAs, and reported outcomes. However, for the RCTs that provided analyzable outcomes, data were pooled and expressed as mean difference (MD) for continuous outcomes and risk ratio for dichotomous outcomes with 95% CIs using a random-effects model to incorporate expected heterogeneity with the ReviewManager program (version 5.3; The Cochrane Collaboration).

In addition, a structured analysis of the outcomes was conducted to derive conclusions regarding the effectiveness and feasibility of CAs for weight management [34]. The CA was regarded as effective if a statistically significant (*P*<.05) improvement was reported in a given outcome. If no significance or significant worsening was reported, the CA was regarded as having no significant evidence supporting it. User experience, such as usefulness, helpfulness, or satisfaction, was rated as positive, mixed, neutral, or negative [35].

## Results

### Study Selection

**Figure 1:**
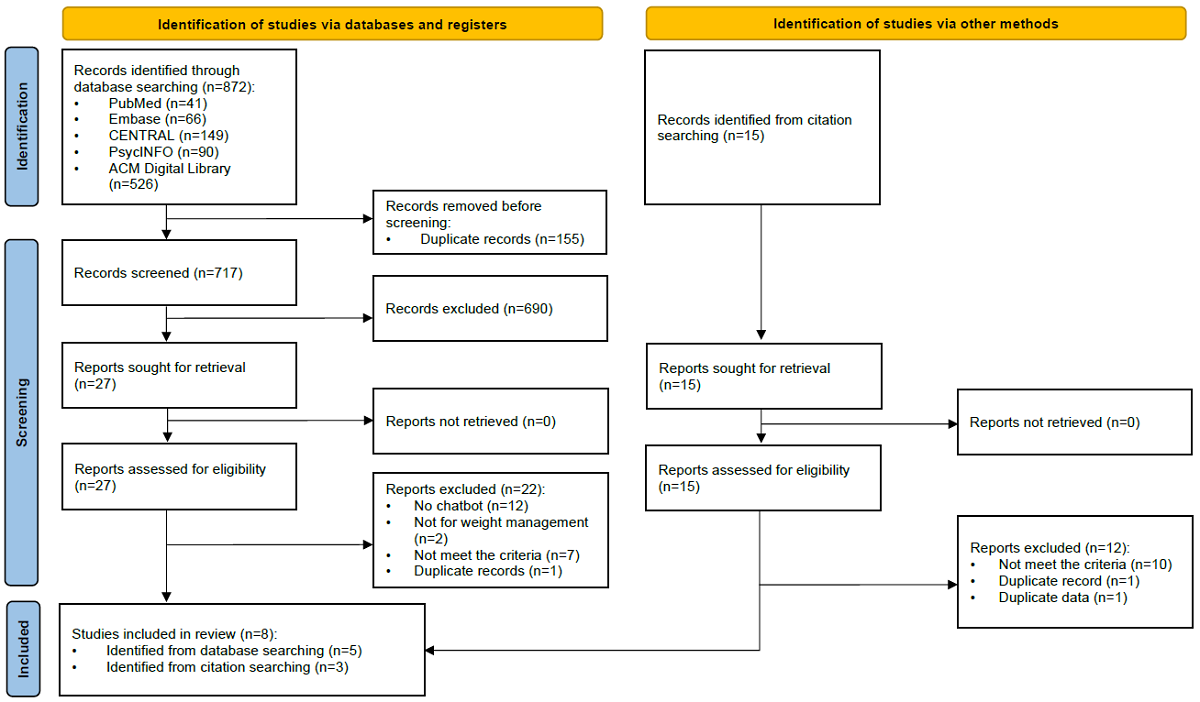
A flow diagram of the literature search for the included studies.

The initial electronic database search yielded 872 citations, and a further 15 studies were identified by hand searching the reference lists of relevant studies. After identifying and removing 17.9% (159/887) of duplicates, the titles and abstracts of the remaining 728 studies were screened, and 95.1% (692/728) of the studies were excluded. The remaining 36 studies were then read in full and excluded if applicable, leaving the final 8 (22%) studies from 9 publications. One of the studies that were finally excluded involved CAs without natural language input capability [36], and another did not use CAs for the purpose of interacting with users in dialogue [37]. The detailed process of study selection is presented in the PRISMA flow diagram (Figure 1).

### Characteristics of the Included Studies

The 8 included studies were conducted in the United States (5/8, 63%), Australia (2/8, 25%), and South Korea (1/8, 13%). The authors of the included studies were mostly related to psychology, medicine, and computer science, that is, the fields of research in each study were as follows: psychology, neuroscience, and biological science [38]; public health [20]; psychotherapy, AI, and pediatrics [39]; exercise and health sciences [40,41]; human-centered design and engineering [42]; informatic and nursing [43]; and physical activity research [44]. The included studies were published in journals of various categories in the Journal Citation Reports: nutrition and dietetics [40], experimental psychology [38], public, environmental and occupational health [39], computer networks and communications [42], and medical informatics [20,41,43,44]. The study participants were recruited from universities [38], primary care [20,39,40], or the web [41-44]. CA interventions were delivered on the web or in a controlled laboratory under the supervision of research assistants [38] but mostly in the participants’ homes [20,39-44]. There were no studies that looked into the use of CAs for weight maintenance, and none of the included studies reported follow-ups after the intervention. The study designs were RCTs (3/8, 38%) [38,40,43] and uncontrolled before-and-after studies (5/8, 62%) [20,39,41,42,44]. In 33% (1/3) of the RCTs, the obesity prevention tutorial was compared with 5 control tutorials using the same intelligence technique, each with slightly different methods of providing the same information on health topics other than obesity or weight management, but the 5 control groups were combined as a single control group as there was little difference in outcomes among them [38]. In another RCT, youth-parent dyads with obesity using CAs were compared with a waitlist group [40], and the other RCT tested the CA versus the same CA features without positive feedback during the first 4 weeks [43]. The other 63% (5/8) of the studies were all single-arm before-and-after comparison studies [20,39,41,42,44].

The number of study participants varied from 23 [39] to 220 [38], with 712 in total in all the included studies. The participants were school-age youths and adolescents (aged 9.0-18.5 years) [39,40], graduate students (mean age 19.2 years, SD 1.7) [38], and adults from different age groups (aged 18.0-76.0 years) [20,41-44].

In total, 37.5% (3/8) of the studies included BMI in the inclusion criteria—BMI ≥25 kg/m^2^ (ie, overweight/obesity [20]) or youths with obesity with BMI ≥95% for age and sex [39,40]. A total of 62% (5/8) of the studies had no restrictions on BMI [38,41-44], but most of the participants were either overweight or obese [41,44]. In total, 12.5% (1/8) of the studies involved female participants only [38], and the remaining studies included both male and female participants [20,39-44]. The study duration was <16 weeks (ie, short term [11]), and 12.5% (1/8) of the studies tested 1 session of a 90-minute tutorial [38]. The main purposes of the tested CAs included contributing to traditional weight management methods of dietary modification [20,38,40,41] and increasing physical activity [38,41-44]. The characteristics of the included studies that focused on weight management are summarized in Table 1.

**Table 1:** Characteristics of the included studies on conversational agents (CAs) and chatbots for body weight management (N=8).

Study and country	Study design	Participants	Mean (SD) age (years)	Main purpose of CA	Control group	Study duration
Wright et al [40], 2013, United States	RCT^a^	Youth-parent dyads with obesity from primary care (24/26^b^, 29 male and 21 female for youths, and 2 male and 48 female for parents)	10.3 (1.1) for youths and 40.0 (9.1) for parents	Dietary modification and decrease in television time	A waitlist	12 weeks
Stein and Brooks [20], 2017, United States	Uncontrolled before-and-after study	Adults with obesity or overweight from primary care (83^c^; NR^d^)	46.9 (15.8)	Dietary modification	N/A^e^	15 weeks
Brust-Renck et al [38], 2017, United States	RCT	Healthy students interested in weight loss from 2 universities (37/183^b^; all female)	19.2 (1.7)	Dietary modification and increase in physical activity	Tutorials on health topics other than obesity	90 minutes
Kocielnik et al [42], 2018, United States	Uncontrolled before-and-after study	Active Fitbit^f^ users (33; 4 male and 29 female)	36.5 (11.2)	Increase in physical activity	N/A	2 weeks
Stephens et al [39], 2019, United States	Uncontrolled before-and-after study	Youths with obesity from primary care (23; 10 male and 13 female)	15.2 (NR)	Behavioral modification	N/A	10-12 weeks
Maher et al [41], 2020, Australia	Uncontrolled before-and-after study	Inactive adults from the community (31; 10 male and 21 female)	56.2 (8.0)	Dietary modification and increase in physical activity	N/A	12 weeks
Piao et al [43], 2020, South Korea	RCT	Healthy office workers (57/49^b^; 25 male and 32 female/21 male and 28 female^b^)	NR	Increase in physical activity	No positive feedback from CA during the first 4 weeks	12 weeks
To et al [44], 2021, Australia	Uncontrolled before-and-after study	Inactive adults recruited on the web (116; 21 male and 95 female)	49.1 (9.3)	Increase in physical activity	N/A	6 weeks

^a^RCT: randomized controlled trial.

^b^Experimental group/control group.

^c^Of them, 70 were included in the data analysis.

^d^NR: not reported.

^e^N/A: not applicable.

^f^Fitbit: commercial fitness-tracking tool.

### Characteristics of the CAs in the Included Studies

The characteristics of the CAs, including name and type of CA, delivery channel, input or output modality, whether they mimicked practice, theories of CAs, and personalization, were summarized and tabulated (Table 2).

The included studies tested 3 types of CAs: an ECA named GistFit [38]; text-based chatbots called Lark [20], Tess [39], Reflection Companion [42], Paola [41], Healthy Lifestyle Coaching Chatbot [43], and Ida [44]; and an automated interactive voice response system called Healthy Eating and Activity Today [40]. In 38% (3/8) of the CAs, only the computer system could start a conversation [38,40,42], whereas in 50% (4/8) of the CAs, both the users and CAs could start a conversation [20,39,41,43,44]. CAs were delivered via various means in the included studies: web-based [38]; mobile apps [20]; telephone [40]; SMS text messages [41,44]; Multimedia Messaging Services [42]; cloud-based instant messaging platforms; Slack (Slack Technologies) [39,41]; and messenger app–based platforms such as WhatsApp (Meta) [39], Facebook (Meta) [39,44], and KakaoTalk (Kakao Corp), the most popular mobile messaging app in South Korea [43]. In total, 62% (5/8) of the CAs in the identified studies accepted the written language, and the most common output was also written language [20,39,41-44]. A total of 12% (1/8) of the CAs used spoken language for both input and output [40], and another used both written and spoken language when presenting information but only written language in tutorial dialogue [38].

The CAs in the included studies were designed to mimic in-person treatment using AI techniques. In 12% (1/8) of the studies [38], 3 female ECAs designed to mimic one-to-one human tutoring delivered information through conversational language (orally and in writing) and facial expressions while displaying images. They also attempted to reply immediately according to the user’s response, correcting mistakes or encouraging them to continue talking [38]. CAs in the other 25% (2/8) of the studies were designed to imitate health care professionals’ compassionate or empathetic health coaching or consultation by delivering messages of emotional support as well as strategic help with users’ difficulties, such as not losing weight or not feeling good [20], or simulating human therapists tailoring their consultation to each client’s needs [39]. Another 25% (2/8) of the CAs imitated a human health coach of behavior change and personalized support [41,42].

All the CAs in the included studies (8/8, 100%) were developed based on psychological theories, such as social cognitive theory or fuzzy-trace theory [38,40], theories of cognitive behavioral therapy (CBT) [20,39], learning theory [42], or behavior change techniques [20,39,41,43,44]. GistFit used content from EatFit, a social cognitive theory–based goal-oriented in-person tutorial designed to improve healthy nutrition and exercise to prevent obesity [38,45]. It also provided an active learning environment by engaging in dialogue based on fuzzy-trace theory (ie, emphasizing meaningful understanding beyond the surface information) to successfully transform behaviors as a result of learning [38]. The conversations of Healthy Eating and Activity Today incorporated social cognitive theory and 2 evidence-based behavior change programs (the Traffic Light diet and the Student Media Awareness to Reduce Television program) [40]. Lark and Tess were developed by integrating CBT for promoting behavior change and increasing self-efficacy through reflection, respect, support, and partnership [20,39]. Paola delivered behavior change techniques such as goal setting, self-monitoring, and personalized feedback to increase users’ physical activity and adherence to the Mediterranean diet [41]. Reflection Companion used reflective questions based on learning to help users understand and articulate their hidden motives and goals [42]. The Healthy Lifestyle Coaching Chatbot delivered interventions based on the habit formation model, which included a push alarm for performing stair-climbing behavior and intrinsic (inner fulfillment and positive reinforcement) or extrinsic (points and coffee coupons) rewards [43]. Ida was designed using the Capability, Opportunity, and Motivation Behavior model to help users improve their motivation via messages, their abilities via feedback on achievement of goals, and their opportunities via education and activity notifications [44].

Personalization was available for 75% (6/8) of the CAs [20,39,41-44]. Lark delivered the personalized content through user information at the setup point (eg, age, sex, weight, height, and goal weight) [20]. Tess, Paola, and Healthy Lifestyle Coaching Chatbot were customized based on the user’s goal and action plan [39,41,43], and furthermore, Tess could adjust interventions by level of user-reported helpfulness [39]. Reflection Companion was designed to diversify the conversation according to the user’s goals, data on physical activity, or aspects of behavior change [42]. Finally, Ida was personalized by adding user information from the research team [44].

**Table 2:** Characteristics of the conversational agents (CAs) in the included studies.

Study and country	Name of CA	Type	Delivery channel	Input and output modality	Mimicking practice	Theories of CAs	Personalization
Wright et al [40], 2013, United States	HEAT^a^	Interactive voice response	Telephone	Spoken and spoken	NR^b^	Social cognitive theory, TLD^c^, and SMART^d^	No
Stein, and Brooks [20], 2017, United States	Lark	Text-based	Mobile app	Written and written	Health professionals’ empathetic health counseling	DPP^e^ and CBT^f^	Yes (by setting users’ age, sex, weight, height, and goal weight)
Brust-Renck et al [38], 2017, United States	GistFit	ECA^g^	Web browser	Written and written and spoken	One-on-one human tutoring	Social cognitive theory and fuzzy-trace theory	No
Kocielnik et al [42], 2018, United States	Reflection Companion	Text-based	SMS text message or MMS^h^	Written and written	Human coaches of behavior change	Reflection based on learning theory	Yes (by diversifying the conversation using users’ goals, graphs of physical activity, and aspects of behavior change)
Stephens et al [39], 2019, United States	Tess	Text-based	SMS text message, Slack^i^, WhatsApp, or Facebook messenger	Written and written	Health professionals’ empathy and compassion	Evidence-based interventions such as CBT, emotionally focused therapy, or motivational interviewing	Yes (by setting specific goals and targeted behavior)
Maher et al [41], 2020, Australia	Paola	Text-based	Slack	Written and written	Health coaches capable of providing personalized support	Behavior change techniques such as goal setting, problem-solving, self-monitoring with feedback, and social support	Yes (by setting step and diet goals)
Piao et al [43], 2020, South Korea	Healthy Lifestyle Coaching Chatbot	Text-based	KakaoTalk^j^	Written and written	NR	Habit formation model with cue-behavior-reward linkage	Yes (by setting behavioral goals and designing push alarms)
To et al [44], 2021, Australia	Ida	Text-based	Facebook messenger	Written and written	NR	The COM-B^k^ model focusing on capability, opportunity, and motivation	Yes (through the research team adding the user information)

^a^HEAT: Healthy Eating and Activity Today.

^b^NR: not reported.

^c^TLD: Traffic Light diet.

^d^SMART: Student Media Awareness to Reduce Television program.

^e^DPP: Diabetes Prevention Program.

^f^CBT: cognitive behavioral therapy.

^g^ECA: embodied CA.

^h^MMS: Multimedia Messaging Service.

^i^Slack: cloud-based instant messaging platform (Slack Technologies).

^j^KakaoTalk: mobile messenger app.

^k^COM-B: Capability, Opportunity, and Motivation Behavior.

### Critical Appraisal of the Included Studies

The methodological quality of the included studies was evaluated. RCTs [38,40,43] were evaluated using the risk-of-bias tool for randomized trials [32], and uncontrolled before-and-after studies [20,39,41,42,44] were evaluated using the Critical Appraisal Skills Programme checklist for cohort studies [33].

As shown in Multimedia Appendices 3 [38,40,43] and 4 [38,40,43], the overall risk of bias for 100% (3/3) of the RCTs was rated as *some concerns* [38,40] and *high risk* [43], mainly with regard to randomization, blinding, and selective results reporting. A total of 33% (1/3) of the studies [38] were judged as having *some concerns* in the domain of the randomization process as reporting was unclear both for the randomization and allocation concealment methods. In the domain of deviations from the intended interventions, 33% (1/3) of the studies [38] were rated as *low risk* considering that they used an intention-to-treat approach. However, another study [40] was rated as *some concerns* as blinding may not have been feasible because of the nature of the waitlist control group and as the intervention also included an electronic health record behavioral counseling tool used by the primary care clinician during well-child follow-up visits. The other study [43] was rated as having a *high risk* as it excluded eligible trial participants after randomization and did not use an intention-to-treat analysis. In addition, the trial by Piao et al [43] had some concerns regarding missing outcome data—it was not reported why participants dropped out, probably because of nonresponse, so it was not clear whether the reasons why they dropped out depended on the true value. A total of 67% (2/3) of the RCTs [38,40] had some concerns regarding the domain of selective reporting because of a lack of published protocols or clinical trial registration information.

As shown in Multimedia Appendix 5 [20,39,41,42,44], the quality of 100% (5/5) of the uncontrolled before-and-after studies was largely low; of the 12 items, the number of *Yes* answers was between 2 [42] and 6 [20,41,44], mainly regarding confounding factor issues and insufficient follow-up. Although most studies (4/5, 80%) had clear objectives, 20% (1/5) of the studies had so many objectives that it was given *No* on question 1 (*Did the study address a clearly focused issue?*) [42]. Selection bias was likely for 20% (1/5) of the studies, which used a consecutive sample of patients admitted to a children’s hospital [39], and another study where participants from a previous health program were contacted [44]. In total, 40% (2/5) of the studies were assessed as likely affected by measurement bias—self-reporting data for weight loss and dietary intake were used in an open study [20], and the other study did not use validated tools for outcome measurement [39,44]. Most of the studies (4/5, 80%) failed to identify and take into account the important potential confounding factors in the research design or analysis (questions 5a and 5b), and only 40% (2/5) of the studies [20,44] adjusted regression analyses on important confounding variables for weight loss (eg, age, sex, height, and baseline weight). In total, 40% (2/5) of the studies [41,44] reported sample size calculations, but for the remaining studies [20,39,42], *Can’t tell* was given as they did not report power analyses and insufficient information was provided to determine whether the follow-up of participants was complete enough (question 6a). As maximal weight loss is generally considered to occur at 6 months [46], the follow-up period was judged to be insufficient for 100% (5/5) of the uncontrolled before-and-after studies [20,39,41,42,44]. For section B of the results, all studies (5/5, 100%) were evaluated as they failed to report reliable results because of poor reporting or wide CIs [20,39,41,42,44]. Most studies (4/5, 80%) except 1 [20] were given *Can’t tell* or *No* with regard to the applicability of the results to the local population, mainly because of small sample sizes, convenience samples, or unbalanced male to female participant ratios of the samples leading to less generalizability. It was difficult to determine whether the results were consistent with those of other studies or whether they had implications for practice as there were few studies on CAs’ weight loss effects (questions 11 and 12).

### Outcomes of the Included Studies

#### Overview

Table 3 summarizes the outcome reporting for the evaluation of the CAs. Overall, 62% (5/8) of the included studies reported outcomes for ≥3 items for CA evaluation [20,38,40,41,44]. Adherence to CAs (7/8, 88%) was the most frequently reported outcome in the included studies (Table 3).

The effect estimates of the RCTs are listed in Table 4, and more detailed results of the included studies are presented in Multimedia Appendix 6 [20,38-44].

**Table 3:** Summary of the outcome reporting for the assessment of conversational agents (CAs).^a^

Study	Weight loss	Diet	Physical activity	User experience	Adherence	Adverse events	Reported items (n=6), n (%)
Wright et al [40], 2013	0	0	1	1	1	NR^b^	5 (83)
Stein, and Brooks [20], 2017	1	1	NR	1	1	NR	4 (67)
Brust-Renck et al [38], 2017	NR	1	1	NR	NR	NR	2 (33)
Kocielnik et al [42], 2018	NR	NR	0	NR	1	NR	2 (33)
Stephens et al [39], 2019	NR	NR	0	1	1	NR	3 (50)
Maher et al [41], 2020	1	1	1	1	1	1	6 (100)
Piao et al [43], 2020	NR	NR	1	NR	1	NR	2 (33)
To et al [44], 2021	NR	NR	1	1	1	NR	3 (50)

^a^Positive or mixed outcomes were coded as 1, and neutral or negative outcomes were coded as 0.

^b^NR: not reported.

**Table 4:** Effects of conversational agents (CAs) on obesity-related outcomes of the included randomized controlled trials.

Outcomes	Study	Results
Weight change (kg)	Wright et al [40], 2013	For children (n=43): MD^a^ −0.9 (95% CI −2.43 to 0.63); for parents (n=43): MD −1.60 (95% CI −8.93 to 5.73)
Diet	Wright et al [40], 2013	Total calorie intake (kcal per day) change (n=43): MD −214 (95% CI −471.9 to 43.9); total fat intake (mg per day) change (n=43): MD −9.5 (95% CI −24.18 to 5.18)
Physical activity	Wright et al [40], 2013	Screen time (hours per day) change (n=43): MD −2.2 (95% CI −4.32 to −0.08)
Physical activity	Piao et al [43], 2020	SRHI^b^ change (n=106): MD 6.70 (95% CI 3.47 to 9.93)

^a^MD: mean difference.

^b^SRHI: Self-Report Habit Index. The scores range from 7 to 84 points; the higher the score, the higher the habit strength of a particular action.

#### Weight Change

Weight change was reported in 33% (1/3) of the RCTs [40] and 40% (2/5) of the uncontrolled before-and-after studies [20,41]. MD in weight over 12 weeks was −0.9 kg, with no statistically significant difference in comparison with a waitlist control (95% CI −2.43 to 0.63; Table 4) [40]; 2.4 kg (95% CI 0.8-4.0) and 2.4% (95% CI 1.0%-3.8%) reduction of baseline weight after 15 weeks of app use was reported [20]; and an average loss of 1.3 kg (95% CI 0.7-2.5) and waist circumference decrease by 2.1 cm (95% CI 0.7-3.5) at 12 weeks of CA use were reported [41] (Table 4 and Multimedia Appendix 6).

#### Dietary Changes

A total of 33% (1/3) of the RCTs reported that there was no significant difference between the groups in diet change. The CA group’s total calorie intake (MD −214, 95% CI −471.9 to 43.9) and total fat intake (MD −9.5, 95% CI −24.18 to 5.18) were similar to those of a waitlist control [40]. In total, 25% (2/8) of the studies reported a change in meal quality; the proportion of healthy eating increased by 59% (95% CI 28.9%-51.7%), whereas unhealthy eating decreased by 11% (95% CI 5.24%-9.85%) [20]. Over the first 6 weeks, the Mediterranean diet adherence score increased significantly, and during the following 6 weeks, it practically remained at this level (mean change from baseline to 12 weeks=5.7, 95% CI 4.2-7.3) [41]. Finally, a 90-minute interactive tutorial with a CA improved knowledge, comprehension, and behavioral intentions for healthier nutrition [38].

#### Physical Activity Changes

In total, 100% (3/3) of the RCTs reported outcomes associated with physical activity. In 33% (1/3) of the RCTs, the CA group’s screen time (hours per day) considerably decreased (MD −2.2, 95% CI −4.32 to −0.08) when compared with a waitlist control group [40]. In another RCT, the Self-Report Habit Index score (ranges from 7 to 84 points; the higher the score, the higher the habit strength of a particular action) of the CA group was significantly higher compared with that of a control CA group where no positive feedback was given by the CA during the first 4 weeks (MD 6.70, 95% CI 3.47-9.93) [41], and in the other RCT [38], it was reported that engaging in dialogues with the CA increased knowledge and helped the users understand the behaviors that helped them prevent obesity. Furthermore, they reported knowledge and comprehension scores after the intervention that correlated with greater behavioral intentions to perform healthy behaviors (Pearson correlation coefficient: 0.2 to 0.4; *P*<.01) [38].

In the remaining 80% (4/5) of the uncontrolled studies, inconsistent results were reported. Total physical exercise time per week rose by 109.8 minutes (95% CI 1.9-217.7) [41], weekly mean step count rose from 10,133 to 11,165 (*P* value not reported) [42], daily step counts significantly increased by 627 (95% CI 219-1035), weekly total minutes of physical activity increased by 154.2 (3.58 times higher at follow-up than at baseline; 95% CI 2.28-5.63), and participants were also more likely to follow the physical activity guidelines (odds ratio 6.37, 95% CI 3.31-12.27) [44]. Stephens et al [39] reported that progress toward targeted goals and actions increased by 81% of the time (*P* value not reported).

#### User Experiences

User experiences such as satisfaction with or usefulness of the CAs were measured using a predefined survey [20,44] or user-reported form [39,40].

Positive user experiences were reported in 50% (4/8) of the studies. More than 75% of users agreed that the CA was useful and helped them eat healthy foods. When it came to watching less television, 78% of parents agreed that the CA was helpful, but only 35% of children did so [40]. The response rate for the survey was 100%, and high acceptability among users was reported—the satisfaction score; net promoter score, which was the intention to recommend the program to others; disappointment score if the weight loss program was not offered; and health outcome score were 87, 68, 47, and 60, respectively [20]. Helpfulness was reported for 96% of the conversation time with the CA [39]. Approximately 33% of users agreed that the CA was useful in increasing confidence and motivation to participate in regular physical activity [44]. Approximately 25% of users agreed that the CA was useful in overcoming barriers, increasing support, and planning for physical activity [44]. The average usability score for the CA (ranges from 0 to 100; the higher the score, the higher the usability and acceptability) was 61.6 (SD 9.7), with most users scoring the CA as *okay* (78.8%) or *good* (10.6%) [44], whereas 53.1% agreed that the CA was helpful to become more active, less participants (43.4%) reported that they would recommend the CA to others, and 35.4% would continue to use it in the future. Although approximately one-quarter of the participants liked the messages that the CA sent out very much, 43.4% (49/113) thought that the chatbot understood their messages most of the time. Most participants experienced technical issues (93/113, 82.3%) and stopped receiving the chatbot messages at some point during the study (95/113, 84.1%) [44].

Negative user experiences were also reported. Although 79% of users believed that the CA may help them modify their lifestyles, they had disappointing experiences, such as the CA answering questions incorrectly, having limited access, the CA not acting quite like a human, and taking a lot of time [41].

#### CA Adherence

A total of 88% (7/8) of the studies reported CA adherence using different types of measurements, such as retention or dropout rate [38,40,41,43], user response rate to CA messages [20,39,42,44], number of contacts with the CA [39-41], and length of conversations [39,42,44]—the retention rate ranged from 90% [41] to 100% [38], and the dropout rate ranged from 12.3% [43] to 13.3% [40]; the number of conversations was 103 (95% CI 75.0-130) at 15 weeks [20], the number of exchanged messages was 4123 for 10 to 12 weeks [39], there were 462 prompts and 429 follow-ups, there were 829 responses from users at 2 weeks [42], and 6.7 (SD 7.0) messages were sent to the chatbot per week [44]; the average number of contacts with the CA was 14.25 (SD 27.58) for 10 to 12 weeks [41], 9.0 (SD 5.7) at 12 weeks [40], and 6.9 per week (64%; range 1-11) [41]; and the average length of conversations was 12.5 minutes [39], decreasing from 170.1 (SD 31.8) characters in the first week to 138.1 (SD 17.0) characters in the second week [42], and 5.1 (SD 7.4) minutes per day [44]. Regarding attrition, 25 out of 251 participants did not complete the tutorial because of a technical problem [38], 81 out of 239 participants failed to record initial height or weight, another 76 participants failed to provide the last weight, and 13 failed to record conversations with the CA in at least 4 separate weeks [20]. The reason for failure to record was not reported.

#### User Engagement and Outcomes

The more users were engaged with the CA interventions, the better outcomes related to weight control were reported in 62% (5/8) of the studies [20,38,40,41,44]. However, there was no analysis of the relationship between attendance and weight loss in 38% (3/8) of the studies [39,42,43]. Frequent use of the CAs was associated with weight loss; in particular, the number of healthy or unhealthy foods logged was a significant predictor of weight loss (β=−.035, 95% CI −0.039 to −0.031) and weight gain (β=.088, 95% CI 0.068-0.107), respectively [20]. Getting involved more fully in dialogues with the CA was associated with greater knowledge and understanding than not participating or than in the control group (*P* value not reported) [38]. High users had a significant weight loss of 4 kg compared with low users (*P*=.001) [40]. The more actively they engaged with the CAs, the better the outcomes of dietary adherence and moderate to vigorous physical activity (*P*=.26 and *P*=.59, respectively) [41]. More exposure to CAs was associated with better outcomes regarding step counts per day (564, 95% CI 120-1009) and adherence to the physical activity guidelines (odds ratio 6.41, 95% CI 3.14-13.09), but physical activity time (176.6 min per week) did not improve [44].

In another study, the effect of engagement with the CA on outcomes was not tested, but it descriptively reported positive progress toward their goals ≥80% of the time based on interviews with active participants [39].

#### Safety, Quality of Life, and Costs

Only 12% (1/8) of the studies reported adverse effects, showing no adverse events related to participation in CA research [41]. There were no reported outcomes for health-related quality of life or cost-effectiveness (Table 3).

## Discussion

### Principal Findings

This systematic review aimed to critically evaluate the effectiveness of CAs on weight loss and obesity-related outcomes and their feasibility in clinical practice. In total, 33% (1/3) of the RCTs and 40% (2/5) of the uncontrolled studies reported weight loss of 1.3 to 2.4 kg at 12 to 15 weeks, and the results, including physical activity change, positive user experience (eg, satisfaction, usefulness, or helpfulness), and adherence to CAs, largely supported the effectiveness and feasibility of CAs. On the basis of the main findings of this systematic review, the use of CAs is promising for behavior change and engagement in weight management programs even though it is difficult to be certain of their impact on weight reduction at present. CAs with unconstrained natural language input largely rely on psychological approaches and personalized content and support close to interpersonal interventions by human teachers or coaches. However, owing to the paucity of adequately designed and well-conducted controlled trials, further research is warranted to establish the role of CAs with unconstrained natural language input in weight management in clinical practice.

### Comparison With Prior Work

Although it was difficult to make a direct comparison, the weight loss of 1.3-2.4 kg [20] was lower than that achieved by DHIs with frequent in-person counseling, but it tended to be similar or better compared with the weight loss achieved by interventions without a human assistant [11]. In a recently published RCT, CAs with only predefined answer options did not considerably change BMI at 6 months in obese adolescents [47]. Another RCT of CA with constrained conversation for maintaining activity level in overweight adults produced no significant weight changes, and the change in step count in the intervention and control groups did not reach the threshold of significance (2.9% vs −12.8%, respectively; *P*=.07) over 12 weeks [48]. Compared with previous systematic reviews on constrained and unconstrained CAs for promoting physical activity, diet, or weight loss [27], better positive outcomes were reported on user experience and adherence in this systematic review. In a systematic review of CAs for mental health, content tailored to users’ needs and conversation through free-text input rather than constrained answer options were regarded as important points for CA users [23]. SMS text messages for behavior change tended to have a greater impact on weight loss under conditions where the messages were bidirectional, personal, and tailored to clinical needs [13,49]. Although research comparing unconstrained conversational CAs with a fixed response is also needed, this suggests that the unconstrained conversation aspects of CAs, leading to personalization and enhanced interactivity with CAs, may be more likely to contribute to weight loss. This implies that more complex, flexible, and personalized conversation might play a substantial role in weight loss.

With regard to the components of CAs contributing to weight loss, mimicking practices such as human health coaching or empathetic health counseling, theories applied to CAs such as psychological approaches (eg, CBT), LM programs (such as the Diabetes Prevention Program), and personalization (such as diversifying the conversation using users’ goals, graphs of physical activity, and aspects of behavior change) were common.

The CAs in the included studies can be divided into a logging and a health coaching part. Previous studies examining calorie-tracking or physical activity–logging apps without health coaching showed no additional weight loss when compared with a control group [50,51]. This implies that the health coaching component might play a substantial role in weight loss using CAs. In addition, the CAs evaluated in this systematic review were designed to incorporate psychological approaches to behavior change and self-efficacy, which are known predictors of successful weight loss [52]. A meta-analysis of CBT interventions on weight loss showed a mean weight loss difference of −1.7 kg (95% CI −2.5 to −0.9; duration: 10.7 months) in favor of the CBT arm [53]. A meta-analysis of studies using CBT [53] found that CBT plus LM [54-56] and CBT with intensive counseling [57] produced greater weight reduction than other interventions. In another meta-analysis, a significant reduction in body weight of 1.47 kg (95% CI −2.05 to −0.88) was reported in the motivational interviewing group compared with the control groups [58]. Although the weight reduction reported in this systematic review was not superior to that of in-person psychological approaches for weight loss, this implies that the psychological approach might play a substantial role in behavior change for weight loss.

The engagement level of CAs in this review was related to better learning outcomes regarding healthy nutrition and exercise [38] and greater weight loss [20]. This is in line with previous findings that contact hours of LM counseling are a predictor of treatment success [39]. Compared with other DHIs, mobile apps showed a high dropout rate [59], and most users rarely used the app after the first month of the study [12]. The engagement strategies of apps include ease of use, design, feedback, function, ability to customize design, tailored content, and phone features [60]. In addition, high SMS text message frequency was associated with retention in the program [61,62]. In this regard, CAs can have the strengths of feedback, customized content, and tailored messages to promote adherence. However, further research is needed to determine whether CAs show better adherence than other DHIs or what mainly affects better adherence to CA interventions.

### Limitations

In this review, only studies on CAs that used unconstrained natural language input through AI and machine learning were considered to reduce DHIs’ drawbacks such as dehumanization and low adherence. A protocol was developed and registered in the Research Registry database, and this systematic review was conducted as per protocol. However, because of a dearth of well-designed and rigorously conducted RCTs, a firm conclusion on whether CAs for weight management actually help maintain or lower weight in the overweight or obese population could not be drawn. Although a thorough literature search was conducted in a range of databases, including not just core medical databases but also psychology and computing or machinery databases, we cannot be completely certain that other potential studies were not overlooked.

The diverse characteristics of the CAs and outcome measures in each study also precluded drawing a definite conclusion. Self-reported weight reduction cannot be entirely free from reporting bias or inaccuracy, and this would have to be considered when interpreting the results. Another limitation is that few studies reported potential adverse events associated with CAs. Adverse event reporting in CA studies is important as machine learning–based CAs are not completely free from the concerns of the *black box effect*, which can generate unpredictable CA responses [21]. In addition, open-text input in CAs may cause serious privacy issues.

Given the breadth of the reported findings in this review, some may argue that a scoping review would better serve our study indication. Although a scoping review identifies knowledge gaps, scopes a body of literature, clarifies concepts, investigates research conduct, and informs a future systematic review, a systematic review summarizes and critically evaluates the current evidence to answer well-crafted specific questions addressing the feasibility, appropriateness, meaningfulness, or effectiveness of a certain treatment or practice [63]. Considering that the initial purpose of our review was to determine whether CAs were effective for weight management and could be applied to clinical practice, we contend that a systematic review approach better suited our study indication.

### Future Directions

Although CAs have been widely used in various fields, research on CAs with unconstrained natural language input in weight management has only just begun. All included studies in this systematic review were published in the last decade. CAs are generally reported to have a positive effect on healthy behaviors, favorable experiences among users, and better adherence. Although the World Health Organization recommends via the global strategy on digital health 2020-2025 that all DHIs be evaluated and their effectiveness be verified in scientific research [64], it was not easy to find robust evidence because of the paucity of studies, short study duration, and lack of adequate follow-up. Owing to an insufficient number of studies and the high heterogeneity in both the methodologies and results of the included studies, limited pooled effects could be calculated for weight loss outcomes. Future RCTs with larger sample sizes, longer treatment durations, and adequate follow-up are warranted to establish a place for unconstrained CAs in body weight management in clinical practice.

### Conclusions

Although it is impossible to draw firm conclusions on CAs’ effects on weight loss at the moment, their use seems promising for behavior change and active participation in weight management programs. Unconstrained CAs seem to have a potential for effective and patient-centered interventions providing education, advice on food selection, and psychological approaches to body weight management via complex and flexible conversation. These findings warrant future controlled studies examining the use of CAs with unconstrained natural language input as an option for weight management.

## Appendix

Multimedia Appendix 1 The PRISMA (Preferred Reporting Items for Systematic Reviews and Meta-Analyses) 2020 checklist.

Multimedia Appendix 2 Search strategies.

Multimedia Appendix 3 Traffic light plot of the domain-level risk-of-bias assessment of the included randomized controlled trials.

Multimedia Appendix 4 Risk-of-bias assessment of the included randomized controlled trials.

Multimedia Appendix 5 Methodological quality assessment of the uncontrolled before-and-after studies using the Critical Appraisal Skills Programme checklist for cohort studies.

Multimedia Appendix 6 Obesity-related outcomes of the included studies.

## Abbreviations

AI: artificial intelligence
CA: conversational agent
CBT: cognitive behavioral therapy
DHI: digital health intervention
ECA: embodied conversational agent
LM: lifestyle modification
MD: mean difference
PRISMA: Preferred Reporting Items for Systematic Reviews and Meta-Analyses
RCT: randomized controlled trial
