# Supplementary material for: Conversational Agents for Body Weight Management: Systematic Review
Source: J Med Internet Res. 2023 May 26;25:e42238. doi: 10.2196/42238 (PMC10257112; doi:10.2196/42238)
Supplement: Multimedia Appendix 2 [file jmir_v25i1e42238_app2.docx]

## Multimedia Appendix 2. Search strategies

1. Search strategy for PubMed (https://pubmed.ncbi.nlm.nih.gov)

#1 obesity [MeSH Terms]

#2 obes* [Text Word]

#3 overweight [MeSH Terms]

#4 overweight [Text Word]

#5 body weight [MeSH Terms]

#6 body weight [Text Word]

#7 body mass [Text Word]

#8 body mass index [MeSH Terms]

#9 body mass index [Text Word]

#10 bmi [Text Word]

#11 OR/1-10

#12 conversational agent* [Text Word]

#13 conversational system* [Text Word]

#14 dialog system* [Text Word]

#15 dialogue system*[Text Word]

#16 relational agent* [Text Word]

#17 chatbot*[Text Word]

#18 chat bot* [Text Word]

#19 chat robot*[Text Word]

#20 social bot* [Text Word]

#21 chatterbot* [Text Word]

#22 virtual avatar* [Text Word]

#23 nursing avatar* [Text Word]

#24 nurse avatar* [Text Word]

#25 social robot* [Text Word]

#26 chatter bot* [Text Word]

#27 virtual assistant* [Text Word]

#28 virtual coach* [Text Word]

#29 artificial agent* [Text Word]

#30 ai bot* [Text Word]

#31 OR/12-30

#32 #11 AND #31

2. Search strategy for EMBASE

(http://www.embase.com.ssl.openlink.khu.ac.kr:8080)

#1 obesity/exp

#2 obes$/t.w

#3 overweight/exp

#4 overweight/t.w

#5 body weight/exp

#6 body weight/t.w

#7 body mass/t.w

#8 body mass index/exp

#9 body mass index/t.w

#10 bmi/t.w

#11 OR/1-10

#12 conversational agent$/t.w

#13 conversational system$/t.w

#14 dialog system$/t.w

#15 dialogue system$/t.w

#16 relational agent$/t.w

#17 chatbot$/t.w

#18 chat bot$/t.w

#19 chat robot$/t.w

#20 social bot$/t.w

#21 chatterbot$/t.w

#22 virtual avatar$/t.w

#23 nursing avatar$/t.w

#24 nurse avatar$/t.w

#25 social robot$/t.w

#26 chatter bot$/t.w

#27 virtual assistant$/t.w

#28 virtual coach$/t.w

#29 artificial agent$/t.w

#30 ai bot$/t.w

#31 OR/12-30

#32 #11 AND #31

3. Search strategy for the Cochrane library

(CENTRAL, http://lps3.www.cochranelibrary.com.kmclibproxy.khu.ac.kr)

Filters: in trials

#1 MeSH descriptor Obesity explode all trees

#2 obes*: ti,ab,kw

#3 MeSH descriptor Overweight explode all trees

#4 overweight: ti,ab,kw

#5 MeSH descriptor Body Weight explode all trees

#6 (body next (weight or mass)): ti,ab,kw

#7 MeSH descriptor Body Mass Index explode all trees

#8 ((body mass index) or bmi): ti,ab,kw

#9 (#1 OR #2 OR #3 OR #4 OR #5 OR #6 OR #7 OR #8)

#10 conversational agent*: ti,ab,kw

#11 conversational system*: ti,ab,kw

#12 dialog system*: ti,ab,kw

#13 dialogue system*: ti,ab,kw

#14 relational agent*: ti,ab,kw

#15 chatbot*: ti,ab,kw

#16 chat bot: ti,ab,kw

#17 chat robot: ti,ab,kw

#18 social bot: ti,ab,kw

#19 chatterbot: ti,ab,kw

#20 virtual avatar: ti,ab,kw

#21 nursing avatar: ti,ab,kw

#22 nurse avatar: ti,ab,kw

#23 social robot: ti,ab,kw

#24 chatter bot: ti,ab,kw

#25 virtual assistant: ti,ab,kw

#26 virtual coach: ti,ab,kw

#27 artificial agent: ti,ab,kw

#28 ai bot: ti,ab,kw

#29 (#10 OR #11 OR #12 OR #13 OR #14 OR #15 OR #16 OR #17 OR #18 OR #19 OR #20 OR #21 OR #22 OR #23 OR #24 OR #25 OR #26 OR #27 OR #28)

# 30 (#9 AND #29)

4. Search strategy for the American Psychological Association PsycINFO

(https://www.proquest.com/psycinfo/)

#1 noft(obese OR obesity OR overweight OR over-weight OR bmi OR “body mass index”) #2 noft(conversational agent* OR conversational system* OR dialog system* OR dialogue system* OR relational agent* OR chatbot* OR chat bot Or chat robot OR social bot OR social robot OR chatterbot OR chatter bot OR virtual avatar OR nursing avatar OR nurse avatar OR virtual assistant OR virtual coach OR artificial agent OR AI bot)

#3 (#1 AND #2)

5. Search strategy for the Association for Computing Machinery Digital Library (https://dl.acm.org/)

[[Title: obes*] OR [Title: "overweight"] OR [Title: "body weight"] OR [Title: "body mass index"] OR [Title: "bmi"] OR [Abstract: obes*] OR [Abstract: "overweight"] OR [Abstract: "body weight"] OR [Abstract: "body mass index"] OR [Abstract: "bmi"]] AND [[Title: chatbot*] OR [Title: chat bot*] OR [Title: chat robot*] OR [Title: social bot*] OR [Title: social robot*] OR [Title: chatterbot*] OR [Title: chatter bot*] OR [Title: virtual avatar*] OR [Title: nursing avatar*] OR [Title: nurse avatar*] OR [Title: virtual assistant*] OR [Title: virtual coach*] OR [Title: artificial agent*] OR [Title: ai bot*] OR [Title: conversational agent*] OR [Title: conversational system*] OR [Title: dialog system*] OR [Title: dialogue system*] OR [Title: relational agent*] OR [Abstract: chatbot*] OR [Abstract: chat bot*] OR [Abstract: chat robot*] OR [Abstract: social bot*] OR [Abstract: social robot*] OR [Abstract: chatterbot*] OR [Abstract: chatter bot*] OR [Abstract: virtual avatar*] OR [Abstract: nursing avatar*] OR [Abstract: nurse avatar*] OR [Abstract: virtual assistant*] OR [Abstract: virtual coach*] OR [Abstract: artificial agent*] OR [Abstract: ai bot*] OR [Abstract: conversational agent*] OR [Abstract: conversational system*] OR [Abstract: dialog system*] OR [Abstract: dialogue system*] OR [Abstract: relational agent*]]
