# Supplementary material for: Conversational Agents for Body Weight Management: Systematic Review
Source: J Med Internet Res. 2023 May 26;25:e42238. doi: 10.2196/42238 (PMC10257112; doi:10.2196/42238)
Supplement: Multimedia Appendix 3 [file jmir_v25i1e42238_app3.docx]

**Multimedia Appendix 3.** Traffic light plot of the domain-level risk of bias assessment of the included RCTs.

| First author (year) | D1 | D2 | D3 | D4 | D5 | Overall |
| --- | --- | --- | --- | --- | --- | --- |
| Wright (2013) [40] |  |  |  |  |  |  |
| Brust-Renck (2017) [38] |  |  |  |  |  |  |
| Piao (2020) [43] |  |  |  |  |  |  |

| Domains:  D1, Randomization process.  D2, Deviations from the intended interventions.  D3, Missing outcome data.  D4, Measurement of the outcome.  D5, Selection of the reported result.  RCT, randomized controlled trial. |  | Low risk |
| --- | --- | --- |
|  |  | Some concerns |
|  |  | High risk |
