# Supplementary material for: Conversational Agents for Body Weight Management: Systematic Review
Source: J Med Internet Res. 2023 May 26;25:e42238. doi: 10.2196/42238 (PMC10257112; doi:10.2196/42238)
Supplement: Multimedia Appendix 4 [file jmir_v25i1e42238_app4.docx]

**Multimedia Appendix 4.** Risk of bias assessment of the included RCTs.

| **Signalling question** | | **Wright (2013)** [40] | | **Brust-Renck (2017)** [38] | **Piao (2022)** [43] |
| --- | --- | --- | --- | --- | --- |
| **Randomization process** |  | |  | | |
| 1.1 Was the allocation sequence random? | | Y | | NI | Y |
| 1.2 Was the allocation sequence concealed until participants were enrolled and assigned to interventions? | | Y | | NI | PY |
| 1.3 Did baseline differences between intervention groups suggest a problem with the randomization process? | | N | | N | N |
| **Risk of bias judgement** | | **Low** | | **Some concerns** | **Low** |
| **Deviations from intended interventions** |  | |  | | |
| 2.1. Were participants aware of their assigned intervention during the trial? | | PY | | PN | PN |
| 2.2. Were carers and people delivering the interventions aware of participants' assigned intervention during the trial? | | PY | | PN | PN |
| 2.3. If Y/PY/NI to 2.1 or 2.2: Were there deviations from the intended intervention that arose because of the experimental context? | | NI | | NA | NA |
| 2.4 If Y/PY to 2.3: Were these deviations likely to have affected the outcome? | | NA | | NA | NA |
| 2.5. If Y/PY/NI to 2.4: Were these deviations from intended intervention balanced between groups? | | NA | | NA | NA |
| 2.6 Was an appropriate analysis used to estimate the effect of assignment to intervention? | | Y | | Y | N |
| 2.7 If N/PN/NI to 2.6: Was there potential for a substantial impact (on the result) of the failure to analyse participants in the group to which they were randomized? | | NA | | NA | PY |
| **Risk of bias judgement** | | **Some concerns** | | **Low** | **High** |
| **Missing outcome data** |  | |  | | |
| 3.1 Were data for this outcome available for all, or nearly all, participants randomized? | | Y | | Y | N |
| 3.2 If N/PN/NI to 3.1: Is there evidence that result was not biased by missing outcome data? | | NA | | NA | N |
| 3.3 If N/PN to 3.2: Could missingness in the outcome depend on its true value? | | NA | | NA | NI |
| 3.4 If Y/PY/NI to 3.3: Is it likely that missingness in the outcome depended on its true value? | | NA | | NA | N |
| **Risk of bias judgement** | | **Low** | | **Low** | **Some concerns** |
| **Measurement of the outcome** |  | |  | | |
| 4.1 Was the method of measuring the outcome inappropriate? | | N | | N | N |
| 4.2 Could measurement or ascertainment of the outcome have differed between intervention groups? | | N | | N | N |
| 4.3 Were outcome assessors aware of the intervention received by study participants? | | PY | | PN | N |
| 4.4 If Y/PY/NI to 4.3: Could assessment of the outcome have been influenced by knowledge of intervention received? | | N | | NA | NA |
| 4.5 If Y/PY/NI to 4.4: Is it likely that assessment of the outcome was influenced by knowledge of intervention received? | | NA | | NA | NA |
| **Risk of bias judgement** | | **Low** | | **Low** | **Low** |
| **Selection of the reported result** |  | |  | | |
| 5.1 Were the data that produced this result analysed in accordance with a pre-specified analysis plan that was finalized before unblinded outcome data were available for analysis? | | NI | | NI | Y |
| 5.2 Is the numerical result being assessed likely to have been selected, on the basis of the results, from multiple eligible outcome measurements within the outcome domain? | | N | | N | N |
| 5.3 Is the numerical result being assessed likely to have been selected, on the basis of the results, from multiple eligible analyses of the data? | | N | | N | N |
| **Risk of bias judgement** | | **Some concerns** | | **Some concerns** | **Low** |
| **Overall risk of bias judgement** | | **Some concerns** | | **Some concerns** | **High** |

NA, Not Available; NI, No Information; N, No; PN, Probably No; PY, Probably Yes; RCT, randomized controlled trial; Y, Yes.
