# Supplementary material for: Conversational Agents for Body Weight Management: Systematic Review
Source: J Med Internet Res. 2023 May 26;25:e42238. doi: 10.2196/42238 (PMC10257112; doi:10.2196/42238)
Supplement: Multimedia Appendix 5 [file jmir_v25i1e42238_app5.docx]

**Multimedia Appendix 5.** Methodological quality assessment of the uncontrolled before-and-after studies with Critical Appraisal Skills Programme checklist for cohort study.

| **Signalling question** | **Stein (2017)** [20] | **Kocielnik (2018)** [42] | **Stephens (2019)** [39] | **Maher (2020)** [41] | **To (2021)** [44] |
| --- | --- | --- | --- | --- | --- |
| **Section A: Validity of the study result** | | | | | |
| 1. Did the study address a clearly focused issue? | ■ | ■ | ■ | ■ | ■ |
| 2. Was the cohort recruited in an acceptable way? | ■ | ■ | ■ | ■ | ■ |
| 3. Was the exposure accurately measured to minimize bias? | ■ | ■ | ■ | ■ | ■ |
| 4. Was the outcome accurately measured to minimize bias? | ■ | ■ | ■ | ■ | ■ |
| 5. (a) Have the authors identified all important confounding factors? | ■ | ■ | ■ | ■ | ■ |
| 5. (b) Have they taken account of the confounding factors in the design and/or analysis? | ■ | ■ | ■ | ■ | ■ |
| 6. (a) Was the follow up of subjects complete enough? | ■ | ■ | ■ | ■ | ■ |
| 6. (b) Was the follow up of subjects long enough? | ■ | ■ | ■ | ■ | ■ |
| **Section B: What are the results?** | | | | | |
| 7. What are the results of this study? |  |  |  |  |  |
| 8. How precise are the results? |  |  |  |  |  |
| 9. Do you believe the results? | ■ | ■ | ■ | ■ | ■ |
| **Section C: Will the results help locally?** | | | | | |
| 10. Can the results be applied to the local population? | ■ | ■ | ■ | ■ | ■ |
| 11. Do the results of this study fit with other available evidence? | ■ | ■ | ■ | ■ | ■ |
| 12. What are the implications of this study for practice? | ■ | ■ | ■ | ■ | ■ |
| **Total items with yes (out of 12 items)** | **5** | **2** | **3** | **6** | **6** |

■, Yes; ■, No; ■, Can’t tell.
