# Supplementary material for: Conversational Agents for Body Weight Management: Systematic Review
Source: J Med Internet Res. 2023 May 26;25:e42238. doi: 10.2196/42238 (PMC10257112; doi:10.2196/42238)
Supplement: Multimedia Appendix 6 [file jmir_v25i1e42238_app6.docx]

**Multimedia Appendix 6.** Obesity-related outcomes of the included studies.

| First author (year) | Weight loss (kg) | Other obesity-related outcomes | | | | |
| --- | --- | --- | --- | --- | --- | --- |
|  |  | Diet | Physical activity | User experience | Adherence | Adverse events |
|  |  |  |  |  |  |  |
| Wright (2013) [40] | For children 1.7 (SD^a^ 3.0); for their parents 0.4 (SD 2.9) | Total calory decrease by 202.0 (SD 397.0) kcal/day; saturated fat decrease by 4.5 (SD 7.2) gm/day; no changes in fruits, vegetables, and total fat intake | TV time decreased by 0.7 (SD 2.2) hour/day | Usefulness: for eating healthy foods more than 75% agreed; for watching less TV 78% of parents and 35% of children agreed | Retention rate: 87.5% Mean number of calls: for parents 9.1 (SD 5.2); for children 9.0 (SD 5.7) | NR^b^ |
| Stein (2017) [20] | 2.4 (SD 0.8) | Healthy meals increased by 31%; unhealthy meals decreased by 54% | NR | SS^c^ 87/100; NPS^d^ 47/100; DS^e^ 68/100; HOS^f^ 60/100 | Number of conversations: 103  Number of weight entries: 6.1  Number of meals logged: 68  Use of apps and weight loss:  1) total number of meals logged predicted weight loss (β -0.035, 95% CI^g^: -0.039 to -0.031, P<0.001);  2) number of unhealthy meals logged predicted weight gain (β 0.088, 95% CI: 0.068 to 0.107, P<0.001) | NR |
| Brust-Renck (2017) [38] | NR | Knowledge, comprehension, and behavioral intentions for healthier nutrition increased | Knowledge, comprehension, and behavioral intentions for healthier exercise increased | NR | The more actively engaged in tutorial dialogues, the better learning outcomes achieved | NR |
| Kocielnik (2018) [42] | NR | NR | Step increased from 10,133 to 11,165 counts/week (*P*-value NR) | NR | Number of exchanged messages: 462 prompts and 429 follow-ups, and 829 responses from users  Average length of conversations: decreasing from 170.1 (SD 31.8) characters in the first week to 138.1 (SD 17.0) in the second week  No test for associations between attendance and weight loss | NR |
| Stephens (2019) [39] | NR | NR | Self-reported progress toward the clinician-inputted targeted goals increased by 81% | Helpfulness: 96% of the conversation time | Number of conversations: 12  Number of exchanged messages: 4,123  Average length of conversations: 12.5 min  No test for associations between attendance and weight loss | NR |
| Maher (2020) [41] | 1.3 (95% CI: 0.7 to 2.5) | Mediterranean diet adherence score increased by 5.7 out of 14 (95% CI: 4.2 to 7.3) | Users meeting their step goal varied from 53% to 78% across weeks | Helpfulness: 79% of users agreed  Negative experiences also reported; incorrect answers, limited access, CA that is not like human, and time consuming | Weekly check-in: average 6.9 (64%, range 1-11)  Mean dietary adherence 91%; the more actively engaged in Paola, the better outcomes of dietary adherence and moderate-to-vigorous physical activity (P = .255, .587, respectively) | No |
| Piao (2020) [43] | NR | NR | Intervention group better than control in SRHI^h^ (*P*=.04) | NR | Dropout rate: 12.26% | NR |
| To (2021) [44] | NR | NR | Step counts/day increased by 627 (95% CI: 219-1,035)  Physical activity/week increased by 154.2 min (3.58 times higher at follow-up than baseline, 95% CI: 2.28-5.63)  Adherence to follow the physical activity guidelines increased by 6.37 (OR^i^, 95% CI: 3.31-12.27) | System Usability Scale^j^: okay (78.8%), good (10.6%), and poor (10.6%)  Helpfulness: 35.4% agreed  Usefulness: 54.0 % of users agreed on increased confidence for physical activity participation; 46.9% of users agreed on overcoming barriers for physical activity participation  44.3% of users agreed on planning to be activated  58.4% of users agreed on staying motivated. | Number of messages 6.7 (SD 7.0)/week  Time spent with CA 5.1 (SD 7.4) min/day  More exposure to CA associated with better outcomes  Step counts/day 564 (95% CI: 120-1,009)  Adherence to follow the physical activity guidelines 6.41 (OR, 95% CI: 3.14-13.09)  Physical activity time (176.6 min/week) did not improve. | NR |

^a^MD, mean difference.

^b^SD, standard deviation.

^c^NR, not reported.

^d^SS, Satisfaction Score (the higher, the better).

^e^NPS, Net Promoter Score (the higher, the more likely to recommend the program to others).

^f^DS, Disappointment Score if the weight loss program were not offered (the higher, the more disappointed).

^g^HOS, (self-reported) Health Outcome Score (i.e. proportion of users reporting somewhat better or much better than before).

^h^CI, confidence interval.

^i^SRHI, Self-Report Habit Index scores (ranges from 7 to 84 points, the higher score, the higher habit strength of a particular action).

^j^OR, odds ratio.

^k^System Usability Scale (0 to 100). The higher score, the higher usability and acceptability. Scores classified into 4 groups: excellent (85.58-100), good (72.75-85.57), okay (52.01-72.74), and poor (0-52.00).
